# Regulatory T Cells Developing Peri-Weaning Are Continually Required to Restrain Th2 Systemic Responses Later in Life

**DOI:** 10.3389/fimmu.2020.603059

**Published:** 2021-02-03

**Authors:** Kathryn A. Knoop, Keely G. McDonald, Chyi-Song Hsieh, Phillip I. Tarr, Rodney D. Newberry

**Affiliations:** ^1^ Department of Internal Medicine, Washington University School of Medicine, St. Louis, MO, United States; ^2^ Department of Immunology, Mayo Clinic, Rochester, MN, United States; ^3^ Department of Pediatrics and Molecular Medicine, Washington University School of Medicine, St. Louis, MO, United States

**Keywords:** regulatory T cells, RORγT, Foxp3, weaning, allergy

## Abstract

Atopic disorders including allergic rhinitis, asthma, food allergy, and dermatitis, are increasingly prevalent in Western societies. These disorders are largely characterized by T helper type 2 (Th2) immune responses to environmental triggers, particularly inhaled and dietary allergens. Exposure to such stimuli during early childhood reduces the frequency of allergies in at-risk children. These allergic responses can be restrained by regulatory T cells (Tregs), particularly Tregs arising in the gut. The unique attributes of how early life exposure to diet and microbes shape the intestinal Treg population is a topic of significant interest. While imprinting during early life promotes the development of a balanced immune system and protects against immunopathology, it remains unclear if Tregs that develop in early life continue to restrain systemic inflammatory responses throughout adulthood. Here, an inducible deletion strategy was used to label Tregs at specified time points with a targeted mechanism to be deleted later. Deletion of the Tregs labeled peri-weaning at day of life 24, but not before weaning at day of life 14, resulted in increased circulating IgE and IL-13, and abrogated induction of tolerance towards new antigens. Thus, Tregs developing peri-weaning, but not before day of life 14 are continually required to restrain allergic responses into adulthood.

## Introduction

The prevalence of food allergies, perennial rhinitis, asthma, and eczema have increased dramatically in recent decades, especially among children (1–3). These allergies are partly attributed to the loss or absence of tolerance to environmental antigens, a process largely meditated by Tregs, which express the transcription factor FoxP3. Current focus has turned to how tolerance induced in early life protects from the development of allergic disorders. Children at high risk for allergic disorders exposed to food allergens, such as peanut or egg, between 4 and 12 months of age had reduced risk of food sensitization compared to children avoiding allergens until after 5 years of age (4–7). Along with food allergen avoidance in early life, another risk factor for allergic disorders is antibiotic use in the first year of life, implicating a role for exposure to microbes in early life, and particularly microbes in the gastrointestinal tract, as protecting against allergy (8–10).

Within the spectrum of allergic disorders, IgE-mediated food allergy is particularly concerning due to its increasing incidence and life-threatening anaphylactic response on allergen consumption (11). Such IgE driven immune responses can be initiated and promoted by type 2 helper (Th2) responses, including the hallmark cytokine IL-13, which is predominantly produce by Th2 T cells, and promotes the production of IgE (12). Such responses can be suppressed by Tregs, therefore understanding the role of Tregs in pathogenesis of food allergy is a key to understanding how prevention of allergic disorders is best maintained.

Exposure to dietary allergens in the first year of life offers protection from future food allergies, while allergen avoidance until after five years of age is linked with increased food allergies (5–7, 13–15). These epidemiologic data are consistent with oral tolerance, or suppression of systemic responses to antigens and allergens first encountered in the gastrointestinal tract (16), having unique features when induced in early life (17). Induction of oral tolerance is correlated to the initiation of regulatory T cells that can suppress Th2 responses (18–20).

A population of Tregs expressing the transcription factor RORγt+ differentiate early in life in a process driven by the microbiota, and may have unique capacities to avoid immunopathologies and restrain Th2 responses (21, 22). Intriguingly this population of Tregs are reduced in children with food allergies (23). Children with food allergies had distinct microbiotas from healthy children, which induced significantly less RORγt+ Tregs (23) suggesting specific microbiota cues during early life are necessary for the development of this population of Tregs. Specific deletion of all Foxp3+ Tregs developing in early life increased gut inflammation, though the phenotype of the Tregs depleted was not determined (24). Moreover, reduced exposure to luminal antigens in early life decreased development of RORγt+ peripheral Tregs (pTregs) and was associated with an increase in Th2 responses to oral antigens (17). Adoptive transfer of RORγt+ pTregs from isolated from the peri-weaning colon reduced Th2 responses against oral antigens suggesting early life RORγt+ pTregs are sufficient to restrain Th2 responses in an unbalanced immune system. Tregs promote tolerance through production of cytokines necessary for the tolerant environment, and therefore Tregs developing in early could contribute to the tolerogenic milieu that gives rise to future Tregs control Th2 responses. However we do not know if these Tregs are continually required to restrain Th2 responses later in life or if their role is restricted to early life to promote the development of a balanced immune system and once developed they are dispensable.

Here we show that the continued presence of “peri-weaning Tregs”, Tregs developing prior to weaning, are necessary for the maintenance and development of tolerance to antigens encountered later in life. We labeled Tregs at weaning with a diphtheria toxin receptor to specifically delete adults of Tregs of early life origin which reduced RORγt+ pTregs, increased serum IgE and IL13, and abrogated tolerance to new orally administered antigens. Thus Tregs developing peri-weaning are a major source of RORγt+ pTregs and are continually required to restrain Th2 responses in later life.

## Materials and Methods

### Mice

All mice were maintained on the C57BL/6 background. C57BL/6 mice, OTII T-cell receptor transgenic mice (25), Foxp3^GFPCreERT2^ mice (26), Rosa^lslDTR^ (27), were purchased from The Jackson Laboratory (Bar Harbor, ME). All mice were fed a routine chow diet. Co-housed littermates were used for experimental controls. All mice were weaned at DOL 21. Adult mice were 8 to 16 weeks of age when analyzed unless stated otherwise. Foxp3^GFPCreERT2^ mice and Rosa^lslDTR^ mice were bred for Foxp3^GFPiDTR^ mice, which were injected with 100µg tamoxifen (Sigma-Aldrich, St. Louis, MO, USA) dissolved in sunflower seed oil with 20% ethanol (Sigma-Aldrich) intraperitoneally (i.p.) on DOL 14 or 24. Mice were then aged to 8 weeks old (DOL 56), and injected with 50µg/kg diphtheria toxin (DT) (Sigma Aldrich) i.p. In some experiments to validate deletion mechanism, Foxp3^GFPiDTR^ mice were injected with tamoxifen on DOL21, and diphtheria toxin (DT) on DOL24. For controls, C57Bl/6 mice were injected with tamoxifen or vehicle (sunflower seed oil with 20% ethanol) on DOL24, and DT on DOL56 to minimize variation due to treatments. Animal procedures and protocols were performed in accordance with the Institutional Animal Care and Use Committee at Washington University School of Medicine.

### Isolation of Cellular Populations and Flow Cytometry

Colons were harvested, rinsed with PBS, and colonic patches were removed. Isolation of splenic and LP cellular populations was performed as previously described (28). Colonic Treg subpopulations were identified as 7AAD^-^, CD45^+^, CD3^+^, CD4^+^, Foxp3^+^, and RORγt^+^. Foxp3 was identified using GFP signal. To detect intracellular antigens (RORγt, cMAF, GATA3, and Tbet) and cytokines (IL13), cells were fixed and permeabilized overnight and stained per the manufacturer’s recommendations (eBioscience). Flow cytometry was performed with a FACScan cytometer (BD Biosciences, San Jose, CA) retrofitted with additional lasers, or an Attune NXT four-laser flow cytometer (Invitrogen). Data acquisition and analysis were performed using Attune NXT software and FlowJo software (Tree Star, Ashland, OR). viSNE analysis was performed with FlowJo software.

### Oral Tolerance and Delayed Type Hypersensitivity Responses

Mice were given Ova 20g/L in drinking water for 10 days beginning at DOL 70. Mice were then immunized subcutaneously with 100µg Ova in incomplete Freund’s Adjuvant (Sigma Aldrich) 14 and 28 days later (i.e., DOL 84 and 98). At 16 weeks of age (DOL 112), mice were challenged with 20 µg Ova in the footpad and 24 h later the DTH response was read as the increase in footpad thickness before and after challenge as measured with micrometer calipers. Body temperature was monitored for one hour following challenge.

### Adoptive T-Cell Transfer of Ova-Specific T Cells

Two days after the start of dietary Ova, mice were injected i.p. with 5 x 10^5^ naïve Ova-specific T cells (CD45.1^+^CD3^+^CD4^+^CD62L^+^Vα2^+^) fluorescence-activated cell sorting (FACS)-isolated from spleens of adult OTII mice on the congenic CD45.1 background. Following transfer, organs were harvested at the indicated time points for cell isolation and analyzed by flow cytometry to detect and evaluate the phenotype of the CD45.1^+^ OTII T-cells. Mice were evaluated seven days after transfer for the phenotype of the transferred CD45.1^+^ OTII T-cells.

### Measurement of Cytokines, and Immunoglobulins

ELISAs specific for IL-13 (eBioscience), Eotaxin (R&D systems), and IgE (eBiosciences) were used per manufacturers recommendations.

### Statistical Analysis

Data were assumed to be normally distributed. Analysis was performed using a Student’s *t* test, one-way ANOVA with a Dunnett’s post-test, or two-way ANOVA with a with GraphPad Prism (GraphPad Software Inc., San Diego, CA) and a two-tailed cutoff of P<0.05 for significance.

### Study Approval

Animal procedures and protocols were performed in accordance with the Institutional Animal Care and Use Committee at Washington University School of Medicine.

## Results

### RORγt+ Tregs Developing Peri-Weaning Persist Into Adulthood

**Figure 1:**
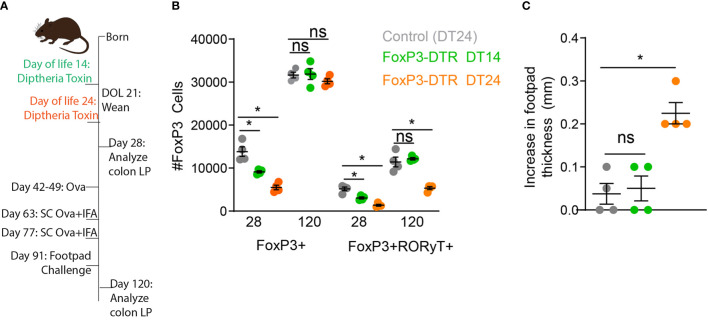
Deletion of Tregs on DOL24, but not DOL14, abrogates oral tolerance later in life. **(A)** Schematic, FoxP3-DTR mice were injected with diphtheria toxin on DOL14 (green) or DOL24 (orange), control mice were injected with diphtheria toxin on DOL24 (gray). **(B)** Absolute number of Foxp3+ or RORγt+FoxP3+ colon LP cells analyzed on DOL 28 or DOL120. **(C)** Increase in footpad thickness following 7 days of Ova in drinking water, immunization, and footpad challenge with Ova, analyzed by one-way ANOVA with Dunnett’s test for multiple comparison. n = 4 mice per group in panels b and c, *denotes statistical significance < 0.05, ns, not significant.

The induction of RORγt+ pTregs begins before weaning and requires exposure to luminal antigens and microbes for their development. We have shown disruption of delivery of luminal substances to the lamina propria during early life reduced development of RORγt+ pTregs (17). This reduction remained statistically significant months later, and was associated with decreased oral tolerance induction and an increase in allergic-type responses against dietary antigens (17). We first asked if specific deletion of FoxP3 cells developing in early life was also associated with decreased oral tolerance. Mice expressing the diphtheria toxin receptor (DTR) exclusively in FoxP3+ cells (Foxp3^DTR^) were injected with the diphtheria toxin (DT) on day of life (DOL) 14 or DOL24 (**Figure 1A**). FoxP3+ cells were significantly decreased on DOL28, in both the DT14 and DT24 groups (**Figure 1B**), but the FoxP3 compartment was restored by DOL120 (**Figure 1B**). Interestingly RORγt+ pTregs remained significantly decreased following DT-depletion of Foxp3 cells at DOL 24, but not at DOL 14 (**Figure 1B**). Thus RORγt+ pTregs develop after DOL 14, in agreement with previous findings (21, 24, 29).

Following deletion of Tregs in early life, we utilized an oral tolerance and delayed-hypersensitivity challenge model by exposing mice to ovalbumin (Ova) in drinking water, immunizing mice with Ova, challenging mice with Ova in the footpad, and measuring footpad swelling as an index of lack of tolerance. Mice had significantly increased footpad swelling, indicating reduced oral tolerance systemically, following deletion of Tregs at DOL24, but not DOL14. Taken along with our previous studies (17), these data highlight the importance of antigen delivery and development of Tregs during early life, but do not address if the RORγt+ pTregs arising during early life are continually required for constraining Th2 responses in later life or if once a balanced immune system is developed these Tregs are dispensable.

**Figure 2:**
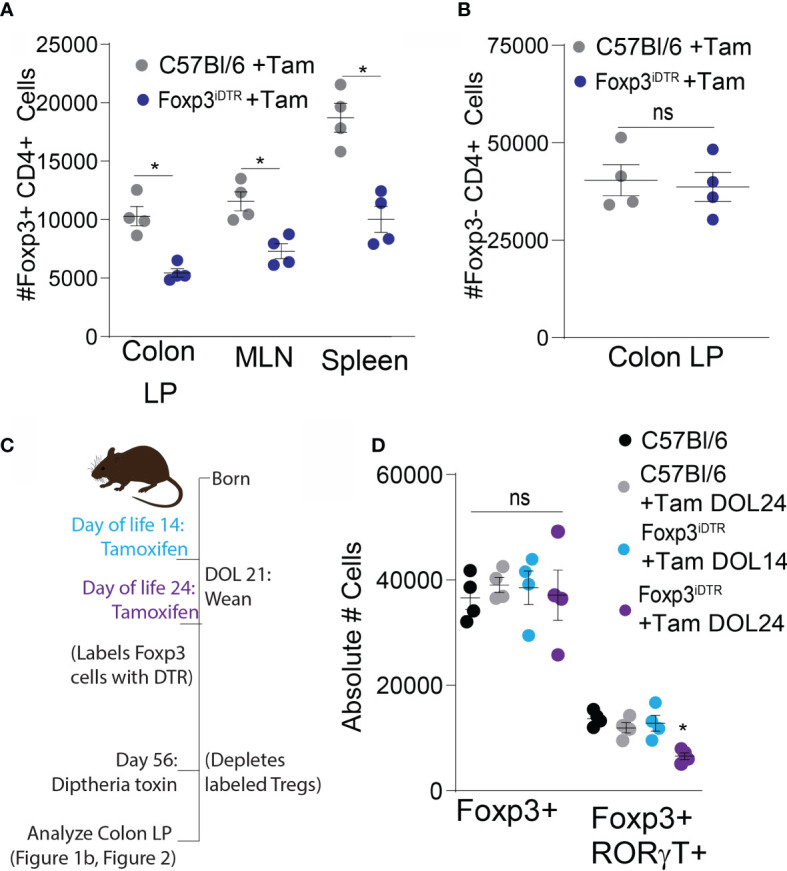
RORγt+FoxP3+ Tregs are induced peri-weaning and are present in the adult colon lamina propria. Control mice (gray) and FoxP3iDTR mice (dark blue) were injected with tamoxifen on DOL21, diphtheria toxin on DOL24, and analyzed on DOL28: **(A)** absolute number of Foxp3+ CD4 T cells and **(B)** absolute number of T cells subsets in colon lamina propria, analyzed by two-tailed Student’s t test. **(C)** Experimental schematic describing labeling of Tregs with DTR using tamoxifen by injecting FoxP3iDTR mice on DOL14 (light blue) or DOL24 (purple). C57Bl6 mice were used as controls and injected with a vehicle (black) or tamoxifen on DOL24 (gray). All groups were injected with diphtheria toxin on DOL56 for deletion of labeled cells on DOL 56. **(D)** Absolute number of Foxp3+ or RORγt+FoxP3+ colon LP cells analyzed on DOL 63 following depletion at DOL 56, analyzed by one-way ANOVA with Dunnett’s test for multiple comparisons. n = 4 mice per group in panels **(A–C)** *denotes statistical significance < 0.05, ns, not significant.

Mice expressing the diphtheria toxin receptor (DTR) under the tamoxifen driven cre in Foxp3 expressing cells, (Foxp3^iDTR^) were used to label Tregs arising at specific times in life to be deleted later. This construct permits the development of a balanced and healthy immune system, including the full Treg compartment and deletion of the Tregs developing in early life to assess their continuing role in later life. For initial validation of this system, Foxp3^iDTR^ and control mice were injected with tamoxifen on DOL21 for labeling with the DTR, which was followed by an injection with DT on DOL24 for deletion of labeled cells. A significant depletion in Foxp3+ cells was quantified on DOL28, indicating DTR was successfully expressed on Foxp3+ cells following tamoxifen labeling in the Foxp3^iDTR^ and that DTR induced deletion of this population (**Figure 2A**), without significantly affecting other T cell subsets (**Figure 2B**).

Next, Foxp3^iDTR^ mice were injected with tamoxifen on DOL 14 or 24, resulting in expression of the DTR in Foxp3+ cells present at those respective points. DT injection on DOL 56 depleted the Tregs that expressed Foxp3 on either DOL 14 or DOL 24 while leaving the Tregs developing after this time unchanged (**Figure 2C**). Depletion of early life Foxp3+ cells in adults resulted in no significant decrease in the total Foxp3+ population in the colon, as the labeled population of Tregs was a small proportion of the total Treg population at DOL 56 (**Figure 2D**). However, there was a significant decrease in the number of RORγt+ pTregs following DT-depletion of Foxp3 cells developing before DOL 24, but not before DOL 14 (**Figure 2D**). Confirming the critical time for RORγt+ pTregs development is after DOL 14 (21, 24, 29), and in our colony largely do not develop after DOL 24. We term all of the FoxP3+ cells developing between DOL14 and DOL24 “peri-weaning Tregs”, which does include the RORγt+ pTregs (17). Tregs developing prior to DOL 14 are predominantly natural thymic derived Tregs and supress autoimmune responses later in life (30).

**Figure 3:**
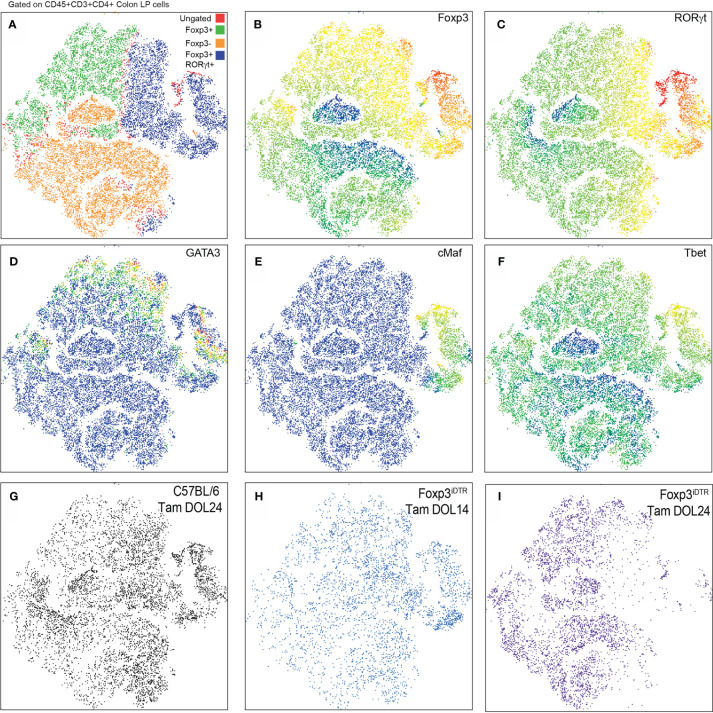
RORγt+FoxP3+ peri-weaning Tregs include a heterogeneous population of Tregs that express GATA3, cMAF, and Tbet. **(A)** viSNE plots of colon CD4 T cell populations from pooled samples denoting Foxp3+ and RORγt+Foxp3+ and Foxp3− clusters. **(B–F)** Plots also show individual expression of Foxp3, RORt, GATA3, cMAF, and Tbet. **(G–I)** Plots of CD4+ T cells in the colon LP from individual experimental conditions. Vsne analysis in **(A–F)** are pooled from n = 3 mice per group, plots in **(G–I)** are representative of n = 3 mice per group.

Multiparameter flow cytometry was performed on the CD4+ T cell population within the colon lamina propria following depletion of Tregs developing prior to DOL14 (Tam DOL14) or peri-weaning Tregs (Tam DOL24) in adults to evaluate the expression of the transcription factors Foxp3, RORγt, cMaf, GATA3, and Tbet. Flow cytometry data was analyzed using a visualization tool for high-dimensional single-cell data based on the t-Distributed Stochastic Neighbor Embedding (t-SNE) algorithm, (viSNE), to visualize cell clusters and loss of cellular populations after deletion. No noticeable cluster was missing from following depletion of Tregs developing prior to DOL14, suggesting such cells either do not cluster into a distinct population, are not long-lived, or that a population with a similar pattern of transcription factor expression is also generated after DOL 14 (**Figure 2**). Depletion of peri-weaning Tregs, those labeled at DOL 24 and depleted in adults, greatly reduced the RORγt+Foxp3+ cluster that included GATA3, cMaf, and Tbet expression (**Figure 3**). Taken together, these data suggest RORγt+ pTregs are largely generated in early life in mice in our colony, that these cell persist into adulthood, and that the peri-weaning Tregs includes a heterogenous population of Tregs expressing diverse transcription factors.

### Peri-Weaning Tregs Restrain Th2 Responses Later in Life

**Figure 4:**
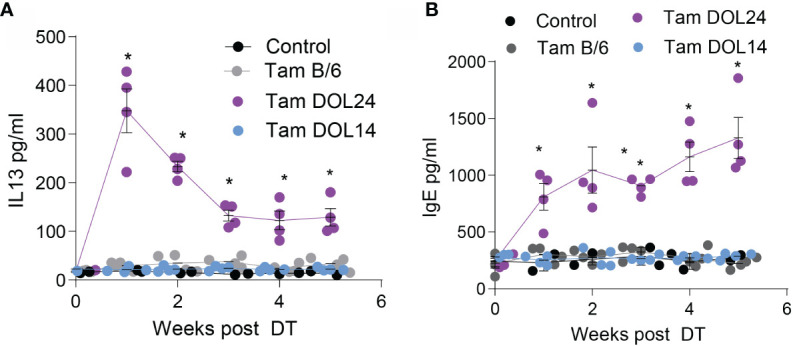
Systemic IL13 and IgE is increased following depletion of peri-weaning Tregs. Serum concentrations of **(A)** IL13 or **(B)** total IgE for 5 weeks following deletion of Tregs. n = 4 mice per group in panels **(A**, **B)**, analyzed by two-way ANOVA with Dunnett’s test for multiple comparisons. * denotes statistical significance < 0.05.

To determine if peri-weaning Tregs were continually required to restrain Th2 responses, mice were monitored following depletion of early life Tregs. Serum IgE and IL13 concentrations were significantly elevated following depletion of peri-weaning Tregs, but not Tregs developing prior to DOL14 (**Figures 4A, B** **)**. Serum IL-13 concentrations initially spiked following depletion of peri-weaning Tregs, but remained significantly elevated throughout the monitoring period. Serum IgE concentrations continued to increase suggesting peri-weaning Tregs are necessary for restraining systemic Th2 cytokines and antibodies.

**Figure 5:**
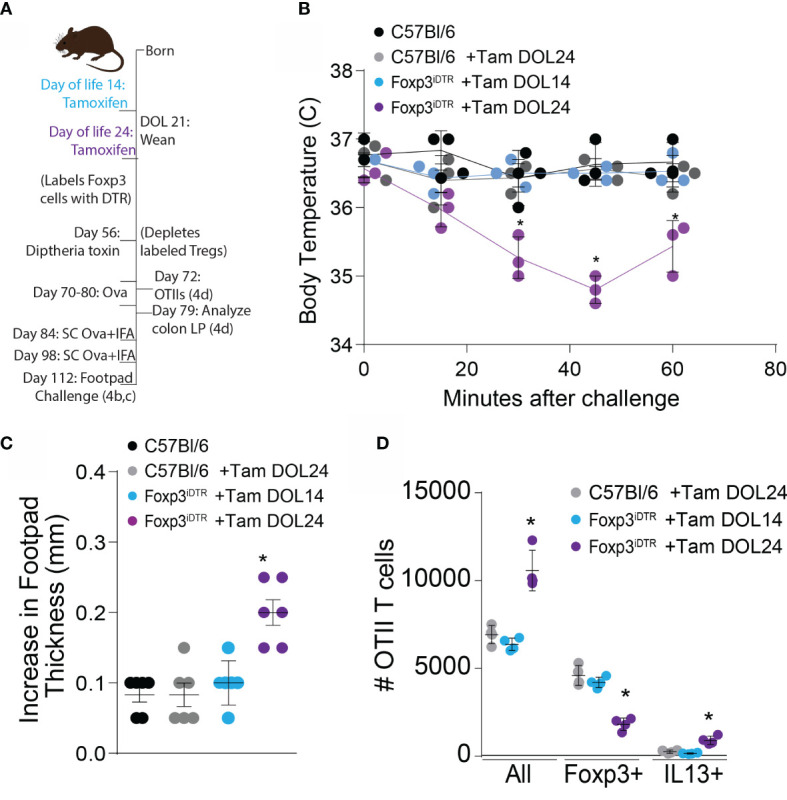
Development of tolerance to new oral antigens is abrogated following depletion of peri-weaning Tregs. **(A)** Experimental schematic describing labeling of Tregs with DTR using tamoxifen followed by deletion of labeled cells on DOL 56, and DTH response (left, **B** and **C**) or transfer of OTII cells (right, **D**) FoxP3iDTR mice were injected with tamoxifen on DOL14 (light blue) or DOL24 (purple). C57Bl6 mice were used as controls and injected with a vehicle (black) or tamoxifen on DOL24 (gray). All groups were injected with diphtheria toxin on DOL56 for deletion of labeled cells on DOL 56. **(B)** Increase in footpad thickness following 7 days of Ova in drinking water, immunization, and footpad challenge with Ova, analyzed by one-way ANOVA with Dunnett’s test for multiple comparison. **(C)** Change in temperature following footpad challenge, analyzed by two-way ANOVA with Tukey’s test for multiple comparisons. **(D)** Number of Ova-specific OTII cells in the colon LP 7 days following adoptive transfer and dietary Ova, analyzed by one-way ANOVA with Dunnett’s test for multiple comparison. n = 6 mice per group in panels **(B, C)** from two independent experiments, c displays data from one representative experiment, n = 3 mice in panel **(D)** * denotes statistical significance < 0.05.

Failure to develop the colonic Treg population in early life spontaneously skewed Th2 profiles that persisted into adulthood and impaired oral tolerance to dietary antigens initially encountered in adulthood. We assessed whether deletion of Tregs developing prior to DOL14 or peri-weaning Tregs in adults impaired oral tolerance to dietary antigens introduced in adulthood by deleting these Tregs in adult mice, exposing mice to ovalbumin (Ova) in drinking water, immunizing mice with Ova, challenging mice with Ova in the footpad, and measuring footpad swelling as an index of lack of tolerance (**Figure 5A**). Mice lacking peri-weaning Tregs had significantly increased footpad swelling, indicating reduced oral tolerance (**Figure 5B**). Additionally, Ova injected into the footpad of mice lacking peri-weaning Tregs caused a decrease in body temperature, suggestive of a hypersensitivity response to the injected antigen (**Figure 5C**).

Ova-specific CD4+ OTII T cells were transferred into mice receiving Ova in drinking water and analysed one week later, to phenotype antigen-specific T cell responses to dietary antigens in mice lacking Tregs developing prior to DOL14 or peri-weaning Tregs. A majority of the OTII T cells became Foxp3+ in the unmanipulated mice and mice depleted of Tregs developing prior to DOL14. In contrast, mice depleted of peri-weaning Tregs had significantly more OTII T cells with reduced Foxp3 expression and increased IL13 expression, suggesting an expansion of effector T cells specific for dietary Ova in the absence of peri-weaning Tregs and a portion of these expressed Th2 cytokines (**Figure 5D**).

## Discussion

Our results demonstrate that continued presence of peri-weaning + Tregs, including RORγt+ pTregs, in adulthood is necessary to restrain Th2 responses and tolerance to dietary antigens encountered initially in adulthood. We have shown that RORγt+ pTregs developing in the colon are longer-lived in the absence of cognate antigen (17). Integrating these observations with prior studies suggests that there are periods in life for the development of Tregs with distinct properties. The first period occurs in the first week of life in mice during which naturally derived Tregs specific for self-antigens develop which function to protect from autoimmunity (30). The next period spanning the interval between the first week through weaning in mice is defined the formation of goblet cell-associated antigen passages (GAPs) in the colon. During this time peripherally induced Tregs (pTregs) specific for non-self-antigens develop extrathymically, a mechanism of Treg induction important for restraint of Th2 responses (31). A portion of these pTregs express the transcription factor RORγt and their development is driven by the microbiota, which is potentially delivered to the colon *via* GAPs (17, 32). The RORγt+ pTregs developing during this period are longer-lived than those developing in adulthood and have the capacity to restrain Th2 responses and promote tolerance to new antigens throughout life. While RORγt+ pTregs can develop throughout life, our data suggest that the RORγt+ pTregs developing after weaning are insufficient to restrain immunopathology.

Intriguingly, peri-weaning Tregs, which includes RORγt+ pTregs, restrained future responses in our model in a non-antigen specific manner suggesting here Tregs act in an innate manner to shape and promote a tolerogenic environment. Within the context of food allergy, the reduction or absence of RORγt+ pTregs could shift the immune environment, influencing both effector subsets and innate cells, within the intestine, allowing for pathogenesis of allergic reactions. Following depletion of peri-weaning Tregs, IgE steadily increased suggesting IgE-producing plasma cell populations either expand or increase secretion. The role of IgE in food allergy is well described, and we previously showed an association of decreased RORγt+ pTregs to increased allergen-specific IgE (17, 32). However, here it remains to be seen if increased total IgE may contribute to future allergic sensitization (33) or just indicate a skewing of the immune system to favor allergic responses. Similarly, increased serum IL13 suggests expansion or increased activity of Th2 effector cells or innate cells such as eosinophils and basophils. IL13 has been shown to not only drive goblet cell hyperplasia, but also increase allergen delivery across the intestinal epithelium by the formation of GAPs and secretory cell associated passages (SAPs) (34), and been implemented in promoting oral allergen sensitization (35) and food allergy pathogenesis (33). Thus increased systemic IL13 has the potential to increase delivery of food allergens into the lamina propria and sensitize innate cells triggering the pathogenesis of allergic responses. How peri-weaning Tregs restrain IL13 production and secretion through either direct cell contact or indirectly is of great interest. While more data is necessary to understand how RORγt+ pTregs uniquely prevent allergic reactions, it remains clear their development during early life may be imperative to maintaining tolerance throughout life.

While the generation and function of these pTregs is an area of active investigation, multiple features, that may be unique to early life, have been identified to contribute to the development of this RORγt+ pTreg population. There are two key time points for the role of antigen delivery: GAP formation in the colon prior to weaning, and GAP and SAP formation in the small intestine later in life. The former drives RORγt+ pTregs expansion in response to microbial products, while the later has the potential to promote food allergy pathogenesis through increased food allergen delivery (34). Understanding how the antigen delivery process results in two very different outcomes depends on both location (colon as compared to small intestine) and timing (peri-weaning compared to later in life). During the pre-weaning interval in which naïve T cells are stimulated to generate these pTregs, dietary and microbial antigens are delivered to the colon lamina propria *via* GAPs to generate antigen-specific immune responses (17, 32). This process is regulated by maternal ligands present in breastmilk and bacterial components of the maturing microbiota around weaning, most notably epidermal growth factor (17, 32). Maternal antibodies IgA and IgG also present in breastmilk have been shown to promote tolerance, may protect from food allergy pathogenesis in early life, and may help regulate development of RORγt+ pTregs (22, 36, 37). Following weaning, dietary antigens are largely encountered by the immune system in the small intestine, and dietary antigen-specific pTregs developing post-weaning have a limited life span in absence of antigen exposure (38, 39). This mechanism might explain why allergen avoidance in early life does not reduce food allergy. Indeed, complementary diets combined food allergens with breastmilk protect against food allergy later in life (14). These feeding practices could be directing food allergens to the colon during the first year of life to promote long lived tolerance *via* the induction of dietary antigen-specific RORγt+ pTregs.

Another feature supporting the development of this RORγt+ pTreg population is the gut microbiota. The requirement of the microbiota for inducing RORγt+ pTregs has long been appreciated (21, 29). Antibiotic use, particularly in the first year of life, is strongly associated with later life food allergies (40), and would certainly induce microbial dysbiosis (41). Children with food allergies have distinct microbiotas from healthy children. These bacterial communities induced significantly fewer RORγt+ pTregs (23). Additionally Th2 responses have the potential to create feedback loops, depleting the bacterial taxa necessary for Treg development (42). Administration of individual or a consortium of bacteria partly rescued RORγt+ pTregs where such cells were otherwise lacking or decreased (23). However this effect was restricted to specific bacterial taxa, suggesting microbial cues exclusive to the early life microbiota promote the development of this population of pTregs (43, 44). However attempts to manipulate the microbiota as a therapy for allergies have had limited success possibly due to the lack of other unique features present in early life.

Finally, RORγt+ pTregs are likely different depending on the time of life during which the pTregs differentiate. Recently a set point for the RORγt+ pTreg population was described to be defined in early life, shortly after birth (22), and pTregs may have age-dependent fates (45). RORγt+ expression can be driven by bacteria adherent to the intestinal epithelium in adult mice, including in pTregs (46–48), however pTregs induced in this manner are transient and dependent on the continued presence of the microbiota (29). The manner in which adherent bacterial antigens are encountered by the immune system post-weaning differs from how commensal bacterial antigens are encountered pre-weaning (32, 49). Common adherent bacteria have not been observed in the peri-weaning microbiota (50, 51), suggesting other microbial members or luminal factors drive RORγt expression peri-weaning. During differentiation RORγt+ pTregs potentially express Foxp3 first, becoming a Foxp3+ RORγt- intermediate before final differentiation into RORγt+Foxp3+ (52). Intriguingly induction of Foxp3 and RORγt expression may be initiated by differential microbial products (53, 54), potentially sensed directly by the Tregs (23).

Taken together, peri-weaning pTregs including the RORγt+ pTregs subset induced in the colon, are uniquely capable of enduring suppression systemic inflammation later in life. The heterogenous expression of specific transcription factors suggest peri-weaning Tregs can restrain multiple Th-driven inflammatory responses (55–57). One limitation of this study is through the labeling of all Foxp3 expressing cells at a specified time in life, deletion of cells upon diphtheria treatment results in mass deletion of Foxp3 cells present at the specified labeling time point, suggesting the mass deletion of cells from a specified time point may be as important as their specificity or gene expression pattern representative of the Foxp3 population.

It remains unclear how peri-weaning RORγt+ pTregs restrain allergic responses uniquely when compared to other Tregs subsets. Multiple described roles for Tregs in the restraint of allergic responses include producing cytokines that suppress immune responses, modulating antigen presenting cells to prevent antigen presentation, and preventing proliferation of T effector cells (58–60). While our data are consistent with each of these functions and suggest peri-weaning Tregs are continually required to perform their suppressive role, one alternative interpretation is the presence of the RORγt+ peri-weaning pTregs limits or decreases differentiation of future Tregs capable of restraining Th2 responses through active utilization of space and resources in the lamina propria as these are long-lived Tregs (17). Future work should explore if the peri-weaning Treg phenotype can be replicated later in life.

In conclusion, we have shown the continued presence of peri-weaning Tregs is necessary to restrain Th2 responses and supports tolerance to dietary antigens encountered later in life. These results implicate peri-weaning Tregs, which include a substantial population of RORγt+ pTregs, as playing active roles in suppressing potential Th2 responses and maintaining tolerogenic homeostasis. Further these findings underscore the importance of early life immune education for the proper expansion of this population, as Tregs developing post-weaning do not substitute for the peri-weaning Tregs. These data suggest the peri-weaning RORγt+ pTregs have unique capacities and are potentially not replaced without intervention. Thus risk factors for food allergy such as antibiotic use and allergen avoidance may contribute to food sensitization by disrupting this population of peri-weaning pTregs.

## Data Availability Statement

The original contributions presented in the study are included in the article/supplementary material. Further inquiries can be directed to the corresponding authors.

## Ethics Statement

The animal study was reviewed and approved by Institutional Animal Care and Use Committee at Washington University School of Medicine.

## Author Contributions

KK performed sample collection, ELISAs, flow cytometry, tSNE analysis, and data analysis. KK, and KM performed animal breeding, genotyping, and injections. KK, C-SH, PT, and RN designed the experiments, analyzed and interpreted the data, and wrote the manuscript. All authors contributed to the article and approved the submitted version.

## Funding

Supported by grants: DK052574-PIT and RDN, DK097317-RDN, AI131342-RDN, AI1407551-RDN and CSH, AI136515-RDN and CSH, AI112626–SPH and RDN, AI131349-CSH, DK109006-KAK, and DK122187-KAK.

## Conflict of Interest

RN, KK, and KM are inventors on U.S. Nonprovisional Application Serial No. 15/880,658 Compositions and Methods for Modulation of Dietary and Microbial Exposure. PT discloses a financial conflict of interest with MediBeacon Inc (member of their Scientific Advisory Board, consultant, and equity holder), is a consultant to Kallyope Inc., and is a potential recipient of royalties from a patent to test human gut permeability noninvasively.

The remaining author declares that the research was conducted in the absence of any commercial or financial relationships that could be construed as a potential conflict of interest.

## References

[1] Branum AM Lukacs SL Food allergy among U.S. children: trends in prevalence and hospitalizations. NCHS Data Brief (2008) 10:1–8. 19389315

[2] Jackson KD Howie LD Akinbami LJ Trends in allergic conditions among children: United States, 1997-2011. NCHS Data Brief (2013) 121:1–8. 23742874

[3] Gupta RS Warren CM Smith BM Blumenstock JA Jiang J Davis MM The Public Health Impact of Parent-Reported Childhood Food Allergies in the United States. Pediatrics (2018) 142:e20181235. 10.1542/peds.2018-1235 30455345 PMC6317772

[4] Poole JA Barriga K Leung DY Hoffman M Eisenbarth GS Rewers M Timing of initial exposure to cereal grains and the risk of wheat allergy. Pediatrics (2006) 117:2175–82. 10.1542/peds.2005-1803 16740862

[5] Nwaru BI Erkkola M Ahonen S Kaila M Haapala AM Kronberg-Kippila C Age at the introduction of solid foods during the first year and allergic sensitization at age 5 years. Pediatrics (2010) 125:50–9. 10.1542/peds.2009-0813 19969611

[6] Koplin JJ Osborne NJ Wake M Martin PE Gurrin LC Robinson MN Can early introduction of egg prevent egg allergy in infants? A population-based study. J Allergy Clin Immunol (2010) 126:807–13. 10.1016/j.jaci.2010.07.028 20920771

[7] Du Toit G Roberts G Sayre PH Bahnson HT Radulovic S Santos AF Randomized Trial of Peanut Consumption in Infants at Risk for Peanut Allergy. New Engl J Med (2015) 372:803–13. 10.1056/NEJMoa1414850 PMC4416404 25705822

[8] Metsala J Lundqvist A Virta LJ Kaila M Gissler M Virtanen SM Mother’s and offspring’s use of antibiotics and infant allergy to cow’s milk. Epidemiology (2013) 24:303–9. 10.1097/EDE.0b013e31827f520f 23348066

[9] Love BL Mann JR Hardin JW Lu ZK Cox C Amrol DJ Antibiotic prescription and food allergy in young children. Allergy Asthma Clin Immunol Off J Can Soc Allergy Clin Immunol (2016) 12:41. 10.1186/s13223-016-0148-7 PMC4988015 27536320

[10] Ni J Friedman H Boyd BC Mcgurn A Babinski P Markossian T Early antibiotic exposure and development of asthma and allergic rhinitis in childhood. BMC Pediatr (2019) 19:225. 10.1186/s12887-019-1594-4 31277618 PMC6612173

[11] Anvari S Miller J Yeh CY Davis CM IgE-Mediated Food Allergy. Clin Rev Allergy Immunol (2019) 57:244–60. 10.1007/s12016-018-8710-3 30370459

[12] De Vries JE The role of IL-13 and its receptor in allergy and inflammatory responses. J Allergy Clin Immunol (1998) 102:165–9. 10.1016/S0091-6749(98)70080-6 9723655

[13] Koplin JJ Dharmage SC Ponsonby AL Tang ML Lowe AJ Gurrin LC Environmental and demographic risk factors for egg allergy in a population-based study of infants. Allergy (2012) 67:1415–22. 10.1111/all.12015 22957661

[14] Du Toit G Sayre PH Roberts G Sever ML Lawson K Bahnson HT Effect of Avoidance on Peanut Allergy after Early Peanut Consumption. N Engl J Med (2016) 374:1435–43. 10.1056/NEJMoa1514209 PMC12333932 26942922

[15] Du Toit G Katz Y Sasieni P Mesher D Maleki SJ Fisher HR Early consumption of peanuts in infancy is associated with a low prevalence of peanut allergy. J Allergy Clin Immunol (2008) 122:984–91. 10.1016/j.jaci.2008.08.039 19000582

[16] Pabst O Mowat AM Oral tolerance to food protein. Mucosal Immunol (2012) 5:232. 10.1038/mi.2012.4 22318493 PMC3328017

[17] Knoop KA Mcdonald KG Coughlin PE Kulkarni DH Gustafsson JK Rusconi B Synchronization of mothers and offspring promotes tolerance and limits allergy. JCI Insight (2020) 5. 10.1172/jci.insight.137943 PMC7455068 32759496

[18] Chehade M Mayer L Oral tolerance and its relation to food hypersensitivities. J Allergy Clin Immunol (2005) 115:3–12. 10.1016/j.jaci.2004.11.008 15637539

[19] Wawrzyniak M O’mahony L Akdis M Role of Regulatory Cells in Oral Tolerance. Allergy Asthma Immunol Res (2017) 9:107–15. 10.4168/aair.2017.9.2.107 PMC5266108 28102055

[20] Tordesillas L Berin MC Mechanisms of Oral Tolerance. Clin Rev Allergy Immunol (2018) 55:107–17. 10.1007/s12016-018-8680-5 PMC6110983 29488131

[21] Ohnmacht C Park J-H Cording S Wing JB Atarashi K Obata Y The microbiota regulates type 2 immunity through RORγt+ T cells. Science (2015) 349:989–93. 10.1126/science.aac4263 26160380

[22] Ramanan D Sefik E Galván-Peña S Wu M Yang L Yang Z An Immunologic Mode of Multigenerational Transmission Governs a Gut Treg Setpoint. Cell (2020) 181:1276–90.e1213. 10.1016/j.cell.2020.04.030 32402238 PMC7393667

[23] Abdel-Gadir A Stephen-Victor E Gerber GK Noval Rivas M Wang S Harb H Microbiota therapy acts via a regulatory T cell MyD88/RORγt pathway to suppress food allergy. Nat Med (2019) 25:1164–74. 10.1038/s41591-019-0461-z PMC6677395 31235962

[24] Al Nabhani Z Dulauroy S Marques R Cousu C Al Bounny S Déjardin F A Weaning Reaction to Microbiota Is Required for Resistance to Immunopathologies in the Adult. Immunity (2019) 50:1276–88.e1275. 10.1016/j.immuni.2019.02.014 30902637

[25] Barnden MJ Allison J Heath WR Carbone FR Defective TCR expression in transgenic mice constructed using cDNA-based alpha- and beta-chain genes under the control of heterologous regulatory elements. Immunol Cell Biol (1998) 76:34–40. 10.1046/j.1440-1711.1998.00709.x 9553774

[26] Rubtsov YP Niec RE Josefowicz S Li L Darce J Mathis D Stability of the regulatory T cell lineage in vivo. Science (2010) 329:1667–71. 10.1126/science.1191996 PMC4262151 20929851

[27] Buch T Heppner FL Tertilt C Heinen T Kremer M Wunderlich FT A Cre-inducible diphtheria toxin receptor mediates cell lineage ablation after toxin administration. Nat Methods (2005) 2:419. 10.1038/nmeth762 15908920

[28] Mcdonald KG Leach MR Brooke KW Wang C Wheeler LW Hanly EK Epithelial expression of the cytosolic retinoid chaperone cellular retinol binding protein II is essential for in vivo imprinting of local gut dendritic cells by lumenal retinoids. Am J Pathol (2012) 180:984–97. 10.1016/j.ajpath.2011.11.009 PMC3349881 22222225

[29] Sefik E Geva-Zatorsky N Oh S Konnikova L Zemmour D Mcguire AM Individual intestinal symbionts induce a distinct population of RORγ+ regulatory T cells. Science (2015) 349:993–7. 10.1126/science.aaa9420 PMC4700932 26272906

[30] Yang S Fujikado N Kolodin D Benoist C Mathis D Immune tolerance. Regulatory T cells generated early in life play a distinct role in maintaining self-tolerance. Science (2015) 348:589–94. 10.1126/science.aaa7017 PMC4710357 25791085

[31] Josefowicz SZ Niec RE Kim HY Treuting P Chinen T Zheng Y Extrathymically generated regulatory T cells control mucosal TH2 inflammation. Nature (2012) 482:395–9. 10.1038/nature10772 PMC3485072 22318520

[32] Knoop KA Gustafsson JK Mcdonald KG Kulkarni DH Coughlin PE Mccrate S Microbial antigen encounter during a preweaning interval is critical for tolerance to gut bacteria. Sci Immunol (2017) 2. 10.1126/sciimmunol.aao1314 PMC5759965 29246946

[33] Wang M Takeda K Shiraishi Y Okamoto M Dakhama A Joetham A Peanut-induced intestinal allergy is mediated through a mast cell-IgE-FcepsilonRI-IL-13 pathway. J Allergy Clin Immunol (2010) 126:306–316, 316.e301-312. 10.1016/j.jaci.2010.05.017 20624645 PMC2917491

[34] Noah TK Knoop KA Mcdonald KG Gustafsson JK Waggoner L Vanoni S IL-13-induced intestinal secretory epithelial cell antigen passages are required for IgE-mediated food-induced anaphylaxis. J Allergy Clin Immunol (2019) 144:1058–73.e1053. 10.1016/j.jaci.2019.04.030 31175877 PMC6779525

[35] Brandt EB Munitz A Orekov T Mingler MK Mcbride M Finkelman FD Targeting IL-4/IL-13 signaling to alleviate oral allergen-induced diarrhea. J Allergy Clin Immunol (2009) 123:53–8. 10.1016/j.jaci.2008.10.001 PMC4121593 18996576

[36] Mosconi E Rekima A Seitz-Polski B Kanda A Fleury S Tissandie E Breast milk immune complexes are potent inducers of oral tolerance in neonates and prevent asthma development. Mucosal Immunol (2010) 3:461–74. 10.1038/mi.2010.23 20485331

[37] Ohsaki A Venturelli N Buccigrosso TM Osganian SK Lee J Blumberg RS Maternal IgG immune complexes induce food allergen-specific tolerance in offspring. J Exp Med (2018) 215:91–113. 10.1084/jem.20171163 29158374 PMC5748859

[38] Kim KS Hong S-W Han D Yi J Jung J Yang B-G Dietary antigens limit mucosal immunity by inducing regulatory T cells in the small intestine. Science (2016) 351:858–63. 10.1126/science.aac5560 26822607

[39] Kulkarni DH Gustafsson JK Knoop KA Mcdonald KG Bidani SS Davis JE Goblet cell associated antigen passages support the induction and maintenance of oral tolerance. Mucosal Immunol (2020) 13:271–82. 10.1038/s41385-019-0240-7 PMC7044050 31819172

[40] Netea SA Messina NL Curtis N Early-life antibiotic exposure and childhood food allergy: A systematic review. J Allergy Clin Immunol (2019) 144:1445–8. 10.1016/j.jaci.2019.08.001 31415783

[41] Shu SA Yuen AWT Woo E Chu KH Kwan HS Yang GX Microbiota and Food Allergy. Clin Rev Allergy Immunol (2019) 57:83–97. 10.1007/s12016-018-8723-y 30564985

[42] Campbell C Dikiy S Bhattarai SK Chinen T Matheis F Calafiore M Extrathymically Generated Regulatory T Cells Establish a Niche for Intestinal Border-Dwelling Bacteria and Affect Physiologic Metabolite Balance. Immunity (2018) 48:1245–57.e1249. 10.1016/j.immuni.2018.04.013 PMC6260932 29858010

[43] Stefka AT Feehley T Tripathi P Qiu J Mccoy K Mazmanian SK Commensal bacteria protect against food allergen sensitization. Proc Natl Acad Sci U.S.A. (2014) 111:13145–50. 10.1073/pnas.1412008111 PMC4246970 25157157

[44] Feehley T Plunkett CH Bao R Choi Hong SM Culleen E Belda-Ferre P Healthy infants harbor intestinal bacteria that protect against food allergy. Nat Med (2019) 25:448–53. 10.1038/s41591-018-0324-z PMC6408964 30643289

[45] Pratama A Schnell A Mathis D Benoist C Developmental and cellular age direct conversion of CD4+ T cells into RORγ+ or Helios+ colon Treg cells. J Exp Med (2019) 217. 10.1084/jem.20190428 PMC7037252 31685531

[46] Ivanov II Atarashi K Manel N Brodie EL Shima T Karaoz U Induction of Intestinal Th17 Cells by Segmented Filamentous Bacteria. Cell (2009) 139:485–98. 10.1016/j.cell.2009.09.033 PMC2796826 19836068

[47] Lathrop SK Bloom SM Rao SM Nutsch K Lio CW Santacruz N Peripheral education of the immune system by colonic commensal microbiota. Nature (2011) 478:250–4. 10.1038/nature10434 PMC3192908 21937990

[48] Chai JN Peng Y Rengarajan S Solomon BD Ai TL Shen Z Helicobacter species are potent drivers of colonic T cell responses in homeostasis and inflammation. Sci Immunol (2017) 2. 10.1126/sciimmunol.aal5068 PMC5684094 28733471

[49] Ladinsky MS Araujo LP Zhang X Veltri J Galan-Diez M Soualhi S Endocytosis of commensal antigens by intestinal epithelial cells regulates mucosal T cell homeostasis. Science (2019) 363:eaat4042. 10.1126/science.aat4042 30846568 PMC6708280

[50] Ivanov II Frutos RDL Manel N Yoshinaga K Rifkin DB Sartor RB Specific Microbiota Direct the Differentiation of IL-17-Producing T-Helper Cells in the Mucosa of the Small Intestine. Cell Host Microbe (2008) 4:337–49. 10.1016/j.chom.2008.09.009 PMC2597589 18854238

[51] Nutsch K Chai JN Ai TL Russler-Germain E Feehley T Nagler CR Rapid and Efficient Generation of Regulatory T Cells to Commensal Antigens in the Periphery. Cell Rep (2016) 17:206–20. 10.1016/j.celrep.2016.08.092 PMC5051580 27681432

[52] Solomon BD Hsieh C-S Antigen-Specific Development of Mucosal Foxp3+RORγt+ T Cells from Regulatory T Cell Precursors. J Immunol (Baltimore Md 1950) (2016) 197:3512–9. 10.4049/jimmunol.1601217 PMC5101183 27671109

[53] Atarashi K Tanoue T Shima T Imaoka A Kuwahara T Momose Y Induction of colonic regulatory T cells by indigenous Clostridium species. Science (2011) 331:337–41. 10.1126/science.1198469 PMC3969237 21205640

[54] Atarashi K Tanoue T Oshima K Suda W Nagano Y Nishikawa H T induction by a rationally selected mixture of Clostridia strains from the human microbiota. Nature (2013) 500:232–6. 10.1038/nature12331 23842501

[55] Wang Y Su MA Wan YY An essential role of the transcription factor GATA-3 for the function of regulatory T cells. Immunity (2011) 35:337–48. 10.1016/j.immuni.2011.08.012 PMC3182399 21924928

[56] Wohlfert EA Grainger JR Bouladoux N Konkel JE Oldenhove G Ribeiro CH GATA3 controls Foxp3^+^ regulatory T cell fate during inflammation in mice. J Clin Invest (2011) 121:4503–15. 10.1172/JCI57456 PMC3204837 21965331

[57] Xu M Pokrovskii M Ding Y Yi R Au C Harrison OJ c-MAF-dependent regulatory T cells mediate immunological tolerance to a gut pathobiont. Nature (2018) 554:373–7. 10.1038/nature25500 PMC5814346 29414937

[58] Noval Rivas M Chatila TA Regulatory T cells in allergic diseases. J Allergy Clin Immunol (2016) 138:639–52. 10.1016/j.jaci.2016.06.003 PMC5023156 27596705

[59] Satitsuksanoa P Jansen K Głobińska A Van De Veen W Akdis M Regulatory Immune Mechanisms in Tolerance to Food Allergy. Front Immunol (2018) 9:2939. 10.3389/fimmu.2018.02939 30619299 PMC6299021

[60] Calzada D Baos S Cremades-Jimeno L Cárdaba B Immunological Mechanisms in Allergic Diseases and Allergen Tolerance: The Role of Treg Cells. J Immunol Res (2018) 2018:6012053–6012053. 10.1155/2018/6012053 30013991 PMC6022267

